# Measuring Plasma Membrane Protein Endocytic Rates by Reversible Biotinylation

**DOI:** 10.3791/1669

**Published:** 2009-12-23

**Authors:** Luke Gabriel, Zachary Stevens, Haley Melikian

**Affiliations:** University of Massachusetts Medical School

## Abstract

Plasma membrane proteins are a large, diverse group of proteins comprised of receptors, ion channels, transporters and pumps.  Activity of these proteins is responsible for a variety of key cellular events, including nutrient delivery, cellular excitability, and chemical signaling.  Many plasma membrane proteins are dynamically regulated by endocytic trafficking, which modulates protein function by altering protein surface expression.  The mechanisms that facilitate protein endocytosis are complex and are not fully understood for many membrane proteins.  In order to fully understand the mechanisms that control the endocytic trafficking of a given protein, it is critical that the protein s endocytic rate be precisely measured.  For many receptors, direct endocytic rate measurements are frequently achieved utilizing labeled receptor ligands.  However, for many classes of membrane proteins, such as transporters, pumps and ion channels, there is no convenient ligand that can be used to measure the endocytic rate.  In the present report, we describe a reversible biotinylation method that we employ to measure the dopamine transporter (DAT) endocytic rate.  This method provides a straightforward approach to measuring internalization rates, and can be easily employed for trafficking studies of most membrane proteins.

**Figure:**
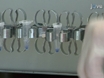


## Protocol

### Procedure Overview:

Using this approach, cell surface proteins are covalently labeled with biotin on available extracellular lysine residues using a membrane impermeant, disulfide-coupled biotinylation reagent (sulfo-NHS-SS-biotin) under trafficking restrictive conditions (i.e. low temperature) (see Fig. 1 for illustration).  One set of cells is shifted to trafficking permissive conditions (37°C) and biotinylated proteins internalize.  The other set of cells are kept at low temperature as controls for 1) the total surface protein at time=0, and 2) stripping control.  Following a short period of internalization, cells are shifted back to low temperature to stop internalization, and any residual surface biotin is stripped off by treating cells with a reducing agent, which cleaves the disulfide-coupled biotin.  Biotinylated proteins that arose from the cell surface and were internalized are protected from the stripping step, and will be the only biotinylated proteins that remain.  Following cell lysis, biotinylated proteins are isolated by streptavidin affinity chromatography and the protein of interest is detected by quantitative immunoblotting. To determine the endocytic rate, the amount of internalized protein is compared to the total surface control labeled at time=0.  We have successfully used this approach to measure the internalization rate of the neuronal norepinephrine^1^ and dopamine^1-4^ transporters.

### Detailed Protocol:

#### Day 1:

1. Plate cells in 6 well plates such that they will be ~80% confluent on Day 2.  Alternatively, if transfected cells are being used, transfect at a density such that they will be ~80% confluent at the time the internalization rate will be measured.  If cells are not strongly adherent, tissue culture ware should be treated with a cell adhesion substrate (e.g. poly-D-lysine) to prevent cell loss during the extensive wash steps.
2. For each protein being tested, plate 2 wells on one plate to be used as the total surface protein (t=0) and stripping controls.  On a second plate, plate one well for each internalization condition being tested (i.e. basal endocytic rate vs. drug-treated).
3. Prepare the following solutions and store at the indicated temperatures for use on Day 2:
  - PBS^2+^: Phosphate buffered saline (pH 7.4) supplemented with 1.5 mM MgCl_2_, 0.2 mM CaCl_2_, (4°C)
  - Biotinylation Quench Solution: PBS^2+^ supplemented with 100 mM glycine, (4°C)
  - NT buffer: 150 mM NaCl, 1.0 mM EDTA, 0.2% BSA, 20 mM Tris, pH 8.6, (4°C)
  - RIPA buffer: 10 mM Tris, pH 7.4, 150 mM NaCl, 1.0 mM EDTA, 0.1% SDS, 1.0% Triton X 100, 1.0% sodium deoxycholate, (4°C)
  - Sulfo-NHS-SS-biotin stock solution:  Dissolve in dimethylsulfoxide (DMSO) to 200 mg/ml, (-20°C)
  - Tris(2-Carboxyethyl) phosphine Hydrochloride (TCEP) stock solution:  500 mM in H_2_O, (-20°C, covered in foil to block light)

#### Day 2:

1. Prepare PBS^2+^ supplemented with 0.18g/ml glucose, 0.2% IgG/protease-free bovine serum albumin (PBS^2+^/g/BSA).  Pre-warm this solution to 37°C in water bath.
2. Thaw out the sulfo-NHS-SS-biotin stock solution on the bench top to melt the DMSO.  Immediately prior to use, prepare fresh sulfo-NHS-SS-biotin solution (2.5 mg/ml in ice cold PBS^2+^, sufficient for 0.75 ml/well).  Vortex the solution vigorously to solubilize the DMSO.  Note that the NHS-biotin reagent is easily hydrolyzed in aqueous solution.  Therefore, all solutions should be prepared immediately prior to use.
3. Biotinylation: Place plates on an ice bath in the cold room and rinse 3 x 2 ml with ice cold PBS^2+^.  Be certain that plates are slightly angled to allow for complete drainage and removal of the buffer solution.  Add 0.75 ml/well of the fresh sulfo-NHS-SS-biotin solution to each well.  Incubate x 15', 4°C on the ice bath with vigorous shaking.  After the incubation is complete, prepare another fresh sulfo-NHS-SS-biotin solution.  Replace the old solution with the fresh solution and incubate x 15', 4°C.
4. Quenching:  It is critical that all non-reacting NHS-biotin molecules are quenched, so that they will not react with and biotinylate intracellular proteins once the cells are lysed.  Wash cells 3 x 2 ml with quenching solution and incubate twice in 2 ml quench solution x 15', 4°C with gentle shaking.
5. Internalization:  If drug treatments are being tested, add appropriate drug concentration to PBS^2+^/g/BSA. Keep control plate at 4°C and bring internalization plate out of the cold room.  Wash 3 x 2 ml with pre-warmed PBS^2+^/g/BSA (+/- drugs) and leave in same solutions (2 ml/well).  Fill any empty wells with pre-warmed solution to ensure even temperature across the plate.  Transfer the cells to a 37°C incubator for 10'.  Immediately prior to end of 37°C incubation, prepare fresh 50 mM TCEP solution in NT buffer to be used for stripping step and store on ice. Prepare sufficient amount for 1.0 ml/well.
6. Stripping: Immediately transfer plate(s) to ice bath and return to cold room.  Rapidly wash cells with ice cold NT buffer, 3 x 2 ml to stop endocytosis.  Also wash strip control wells 3 x 2 ml with NT buffer.  Add 1.0 ml fresh stripping solution to each well.  Incubate on ice, 15', 4°C with gentle shaking.  Replace wells with fresh stripping solution and incubate an additional 15', 4°C on ice.
7. Lysis: Wash all wells that were exposed to stripping solution, 3 x 2 ml NT buffer.  Then wash all wells (including total controls) with 3 x 2 ml PBS^2+^.  Lyse in 300μl/well RIPA buffer (or other lysis buffer compatible for the protein of interest) containing fresh protease inhibitors (1.0 mM PMSF, 1.0 μg/ml each leupeptin, aprotinin and pepstatin).  Lyse by shaking x 20', 4°C.  Transfer to microfuge tubes and clear cellular debris by centrifuging 18,000 x g, 10', 4°C.
8. Protein concentration determination: Use a protein assay compatible with your lysis conditions (e.g. *DC* protein assay, Bio-Rad) to determine the protein concentration of the lysates as compared to a standard BSA curve.
9. Streptavidin affinity chromatography: Prepare microfuge tubes with equivalent amounts of protein for each sample.  Add lysis buffer to bring final volume of each sample to 200μl.  Vortex the streptavidin agarose beads vigorously to bring to an even suspension.  Using a 200μl pipettor with the tip cut off, pipette beads into each tube.  Recommended 20μl beads/50 μg lysate.  Incubate overnight, 4°C on a tube rotator.

#### Day 3:

1. Bead washing: Centrifuge samples, 18,000 x g, 2' to collect beads.  Aspirate off lysate, being careful not to approach bead pellet with aspirator.  Use a plastic pipette tip on the end of the aspirator for better control.  Add 0.75 ml lysis buffer to each tube, and vortex to wash beads. Centrifuge samples, 18,000 x g, 2' to collect beads and repeat aspiration and washing twice (three washes in total).  After the final wash, aspirate as much of the buffer as possible without disturbing the bead pellet.  Tipping the tube toward the aspirator assists with this step.
2. Sample elution: Add 20-25μl 2x Laemmli sample buffer (reducing) to each tube.  The reducing agent will cleave the NHS-SS-biotin disulfide bond, releasing the isolated proteins into solution.  Most membrane proteins are highly vulnerable to aggregation when boiled, and should not be heated prior to loading on gels for SDS-PAGE.  Determine heat sensitivity of your protein of interest prior to performing these experiments.  If protein cannot tolerate boiling/heating, incubate on a rotator, 30', room temperature prior to analyzing by SDS-PAGE.  This is sufficient to cleave the disulfide bond and elute the proteins.
3. SDS-PAGE and immunoblotting: Separate proteins by SDS-PAGE.  For each sample, it is easiest to run the samples in the following order:  total surface (time=0), strip, internalization condition#1, Condition#2, etc.  Transfer to membrane for immunoblotting and blot with appropriate antibody for your protein of interest.  Capture immunoreactive bands using a CCD-camera gel documentation system, being certain that there are no saturated pixels.  Quantify each band density using gel analysis/densitometry software.

### Representative Results:

A representative immunoblot result is shown in Figure 2A.  The strongest signal is in the "total" lane (T), which is total amount of surface protein prior to internalization.  The "strip" control (S) should ideally be close to blank, which demonstrates that the strip was efficient for the experiment.  The strip efficiency is calculated by comparing the density of the "strip" lane to that of the "total" lane (e.g. protein that was biotinylated in parallel with the strip, but was neither warmed to 37°C nor exposed to stripping solution).  The following formula is used:[1-(strip/total)]*100%

Using this formula, the strip in Figure 2 was 99.8% efficient.  Finally, you will see bands of lesser intensity than the total in the internalization lane(s) (I).  In the example (Figure 2), cells treated were treated with either vehicle or 1μM phorbol myristate acetate (PMA) during a 10' internalization and dopamine transporter internalization rates were measured for a 10' initial internalization period.  The internalization rate is calculated as follows: internalized/total*100

As seen in Figure 2B, 10.4% surface DAT internalized over 10' under vehicle-treated conditions.  PMA treatment increased DAT internalization rates to 23.2% of total surface DAT.


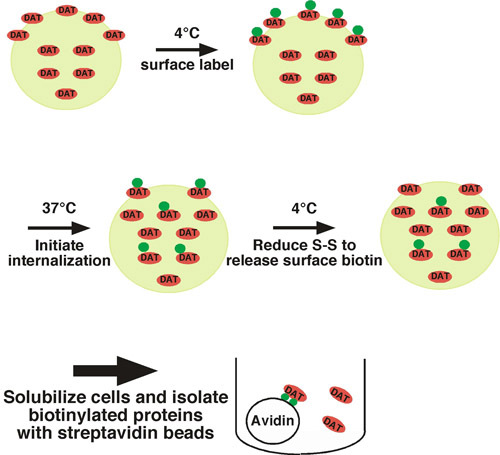
 **Figure 1. Protocol illustration.** Cells are biotinylated at 4°C to exclusively label the surface population, and are shifted to 37°C to initiate internalization.  Following internalization, cells are rapidly chilled to stop endocytic processes and residual surface biotin is stripped by treating cells with a reducing agent.  The only biotinylated proteins that remain are those that arose from the surface at t=0 and were internalized, thus protecting them from the stripping treatment.  Biotinylated proteins are isolated by batch affinity chromatography with streptavidin beads and the protein of interest is detected by immunoblotting.


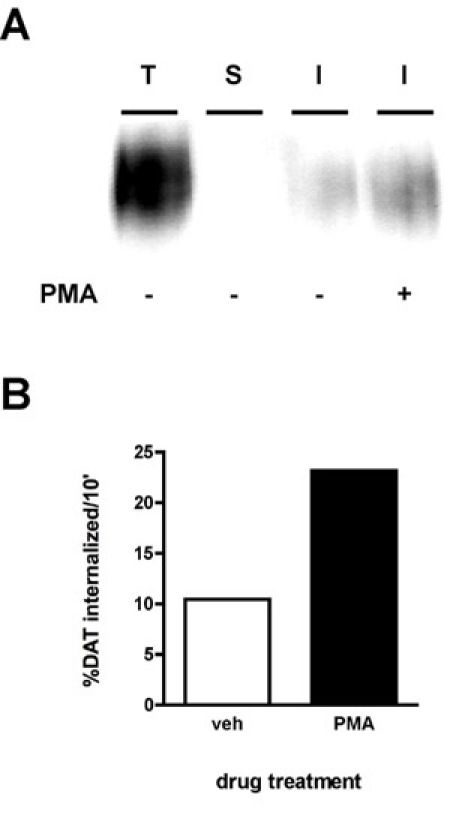
 **Figure 2. PKC activation increases the DAT endocytic rate.** Internalization assay.  PC12 cells stably expressing DAT were biotinylated, 4°C as described in "Detailed Protocol".  Cells were rapidly warmed to 37°C ±1 μM PMA and incubated 10', 37°C.  Residual biotin was stripped by reducing, cells were lysed and biotinylated proteins were isolated by streptavidin affinity chromatography.  (A) Representative immunoblot showing total surface DAT at t=0 (T), strip control (S), and internalized DAT (I) under the indicated conditions.  (B)  Bands were captured with a CCD camera and quantified with Quantity Data software (Bio-Rad).  Data are expressed as % total DAT internalized/10 min.


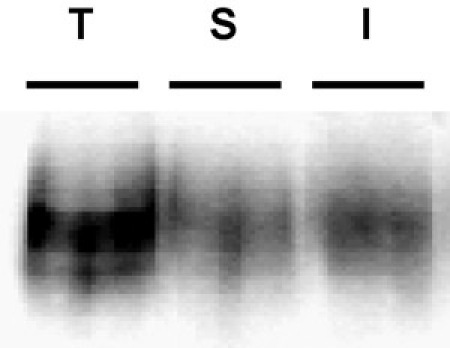
 **Figure 3. Example immunoblot depicting a poor biotin strip.**  Internalization assay. PC12 cells stably expressing DAT were biotinylated, 4°C as described in "*Detailed Protocol*".  Cells were rapidly warmed to 37°C and incubated 10', 37°C.  Residual biotin was stripped by reducing, cells were lysed and biotinylated proteins were isolated by streptavidin affinity chromatography.  The immunoblot shows total surface DAT at t=0 (T), strip control (S), and internalized DAT (I).  Note the visible band in the strip control lane, indicative of poor strip efficiency.

## Discussion

Common problems: The most common problem that arises in these experiments is poor strip efficiency.  The efficiency of the strip is critical in being able to interpret the results.  Unless the strip was highly efficient, it is not possible to conclude that any biotinylated proteins in the internalization lanes were, in fact, internalized from the surface.  Strips ≥90% efficiency are optimal, and we discard any results if the strip falls below this level.  An example of a poor strip is shown in Figure 3.  Note that the band in the strip lane is quite visible, and corresponds to a strip efficiency of 34%.  If poor strip efficiencies occur, fresh TCEP may not have been made immediately prior to adding to the cells.  Alternatively, the TCEP may be degraded and new reagent will need to be purchased. Use of TCEP powder, rather than solution, may circumvent this problem.

Possible modifications: In the current report, we measured a relative DAT internalization rate, taken over the course of the initial 10 minutes of internalization, and compared it to the DAT internalization rate during PKC activation.  Alternatively, an absolute rate could be measured by warming cells for increasing time points.  While this may be possible, our experience suggests that the low signal levels at early time-points, coupled with inter-experimental variability, do not yield highly reproducible results at very early time-points.  Also, although we used adherent cells, the assay can be modified for cell suspensions.  Tissue suspensions, such as synaptosomes, can also be used.  However, it is imperative that the integrity of the membranes is first established.  If the preparation has a significant percentage of "leaky" membranes, the biotinylation reagent will label intracellular proteins and it will not be possible to reliably determine the endocytic rate.  In this situation, immunoblots should also be probed for an intracellular marker protein, such as actin, to test whether the intracellular protein pool was exposed to the biotinylation reagent.
